# An Adaptive, Discrete Space Oriented Wolf Pack Optimization Algorithm for a Movable Wireless Sensor Network

**DOI:** 10.3390/s19194320

**Published:** 2019-10-06

**Authors:** Dongxing Wang, Huibo Wang, Xiaojuan Ban, Xu Qian, Jingxiu Ni

**Affiliations:** 1 School of Mechanical Electronic & Information Engineering, China University of Mining & Technology, Beijing 100083, China; wangdongxing85@163.com (D.W.); xuqiancumtb@163.com (X.Q.); 2 Beijing Advanced Innovation Center for Materials Genome Engineering, University of Science and Technology Beijing, Beijing 100083, China; 3 Engineering Integrated Experimental Teaching Demonstration Center, Beijing Union University, Beijing 100101, China; njx1211@163.com

**Keywords:** wireless sensor network, coverage rate, wolf pack algorithm, optimization, probability matrix, adaptive step size

## Abstract

Recently, many related algorithms have been proposed to find an efficient wireless sensor network with good sustainability, a stable connection, and a high covering rate. To further improve the coverage rate of movable wireless sensor networks under the condition of guaranteed connectivity, this paper proposes an adaptive, discrete space oriented wolf pack optimization algorithm for a movable wireless sensor network (DSO-WPOA). Firstly, a strategy of adaptive expansion based on a minimum overlapping full-coverage model is designed to achieve minimum overlap and no-gap coverage for the monitoring area. Moreover, the adaptive shrinking grid search wolf pack optimization algorithm (ASGS-CWOA) is improved to optimize the movable wireless sensor network, which is a discrete space oriented problem. This improvement includes the usage of a target–node probability matrix and the design of an adaptive step size method, both of which work together to enhance the convergence speed and global optimization ability of the algorithm. Theoretical research and experimental results indicate that compared with the coverage algorithm based on particle swarm optimization (PSO-WSN) and classical virtual force algorithm, the newly proposed algorithm possesses the best coverage rate, better stability, acceptable performance in terms of time, advantages in energy savings, and no gaps.

## 1. Introduction

The wireless sensor network (WSN) is one of the most promising technologies for some real-time applications because of its size, cost-effectiveness, and easily deployable nature [1]. For decades, with the rapid development of the Internet of Things, artificial intelligence, and other advanced technologies, the use of wireless networks as a basic technology has been studied a great deal by researchers, such as in [2], in which Dai et al. proposed a novel multichannel network with infrastructure support (called the MC-IS network), which has not been studied in the literature. To solve the problems of large-scale wireless networks, with their wide variety, high volume, real-time velocity data and huge value, which leads to unique research challenges that are different from existing computing systems, in [3], researchers presented a survey of state-of-the-art big data analytics (BDA) approaches for large-scale wireless networks and the technical solutions to challenges in BDA for large-scale wireless networks according to each stage in the life cycle of BDA. They also discussed open research issues and outlined future directions in this promising area. 

In particular, WSNs are being used for more and more applications, such as in military reconnaissance, environmental monitoring, smart homes, and medical health, and so on; for example, in [4], Thakur et al. reviewed the applicability of wireless sensor networks in precision agriculture to identify various WSN technologies adopted for precision agriculture and the impact of these technologies on achieving smart agriculture. In [5], Li et al. presented a forest fire detection system framework based on WSN and its implementation scheme, then they discussed some key problems, focusing on forest fire forecast modeling, WSN node deployment, WSN nodes and forest fire positioning, and wireless communication control protocol. In [6], WSN was used to monitor the water environment and automatically collect information on the ecological environment, meteorological environment, water pollution, and so on. In [7], because existing opportunity networks can hardly satisfy the requirements of disaster scenarios for low-latency messaging, Fu et al. developed a new opportunistic network framework, the WSN-assisted opportunistic network (WAON), and proposed a forwarding mechanism, net spray for WAON, which supports mobile-to-mobile, static-to-mobile, mobile-to-static, and static-to-static operations.

However, in a complex and changeable working environment, WSNs need a large number of sensor nodes to cooperate with each other to complete detection and sensing tasks [8]. For various monitoring areas, a reasonable arrangement of sensor nodes is conducive to improving the working efficiency and reducing the energy consumption of WSNs. Mobile nodes are used to dynamically reconstruct the network structure, expand the network coverage rate, and optimize performance; thus, they have become one of the key technologies of WSNs [9]. 

Accordingly, in recent years, researchers have put forward many effective methods for solving the location optimization problem of wireless sensor nodes from different perspectives. In [10], Zhou et al. proposed an improved algorithm based on virtual force where the WSN was divided into grids and nodes, which were redeployed under the adaptively chosen resultant force to enable WSN to gain the best coverage. In [11], in order to solve the coverage problem, Zhang et al. proposed a coverage algorithm based on virtual forces including an enhanced coverage algorithm, a connectivity-preserved method for neighboring nodes, and a coverage algorithm for the relevant area. In [12], Yarinezhad et al. proposed a fixed parameter tractable (FPT) approximation algorithm with an approximation factor of 1.2 for the load-balanced clustering problem (LBCP) and an energy-efficient, balanced routing algorithm, which effectively solved the load-balanced clustering problem and maximized the network’s lifetime by reducing the energy consumption. In [13], Chen et al. proposed a novel wireless sensor network with energy-efficient coverage that achieved a good balance between target coverage and energy consumption by fusing the genetic algorithm (GA) and WSN. In [14], Somaieh et al. proposed the distributed energy-aware hexagon-based clustering algorithm to improve coverage (DEHCIC), which considers energy and topological features such as the number of mobile neighbor nodes and number of neighbor nodes to elect cluster heads and attempts to cover holes as much as possible by static sensor nodes, while the closest mobile node is used to cover holes if this is not possible; furthermore, the proposed algorithm retains sensor nodes in an active mode that covers interest points and puts others into a low-power sleep mode. In [15], Alavi et al. proposed a distributed event-triggered control strategy for DC microgrids, based on the publish–subscribe model over industrial wireless sensor networks, to efficiently stabilize grid voltage and to further balance the energy level of energy storage systems. In [16], Sajwan et al. proposed a novel routing algorithm for wireless sensor networks, which achieved uniform energy depletion across all nodes and, thus, led to a prolonged network lifetime. In [17], to improve network coverage, So-In et al. proposed a novel distributed deployment algorithm, the coverage hole-healing algorithm (CHHA), to maximize area coverage. In [18], Wang et al. proposed “parallel particle swarm optimization-based mobile sensor node deployment in wireless sensor networks” by adopting particle swarm optimization (PSO) in a parallel mechanism to optimize the deployment of mobile sensor nodes. 

Unfortunately, all of these algorithms have their own shortcomings, in that some coverage rates are insufficient, some can only be used in specific environments due to constraints, and so on.

In 2007, Yang and co-authors proposed the wolf swarm algorithm [19], which is a new swarm intelligence algorithm. Because of its excellent performance, some special variants of the wolf pack optimization algorithm have been proposed to gain optimal solutions. For example, in [20], Wang et al. proposed “an adaptive distributed size wolf pack optimization algorithm using the strategy of jumping for raids” in order to enhance performance. In [21], by introducing the position order coding method in the initialization phase, Huang et al. studied the path optimization problem in discrete domains and introduced a secondary search in the iterative process to improve the speed and accuracy of the algorithm to determine the optimal solution before the maximum number of iterations. In [22], Wu et al. proposed a discrete wolf pack algorithm by redesigning the position of artificial wolves and intelligent behaviors to solve the traveling salesman problem. In combination with the probabilistic nearest neighbor method, the proposed algorithm preserved the cooperative searching feature based on the job distribution of the wolf pack and was able to balance both the breadth and depth of the searching ability. In [23], a novel, discrete GWO was proposed where a random leader selection was performed, and the probability for the main leader to be selected increased at the detriment of the other leaders across iterations. In [24], a new model based on the discrete wolf pack search (DWPS) algorithm was proposed to maximize the number of satisfied passengers, the total number of transfers, and the total travel time of all served passengers for a transit network design problem.

Consequently, the low coverage of WSN and good performance of the wolf pack optimization algorithm allow us to attempt to fuse the coverage problem between the two. Therefore, on the premise of ensuring adequate coverage and absolute connectivity, this paper proposes a method to find the shortest path to minimize the energy consumption of the whole sensor network.

## 2. Related Works

### 2.1. Adaptive Shrinking Grid Search Wolf Pack Optimization Algorithm (ASGS-CWOA) [25]

The ASGS-CWOA was proposed in 2018 by Wang et al. in [25], and it is an efficient variant of the wolf pack optimization algorithm in the optimization field aimed at solving application problems efficiently. Its key improvements are as follows.

• Method of Adaptive Shrinking Grid Search (ASGS)

**Figure 1:**
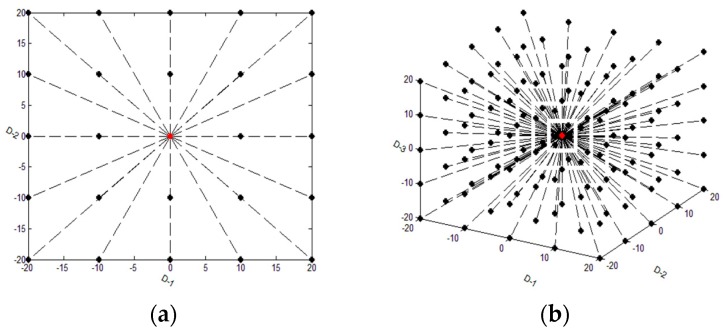
(**a**) The searching situation of wolves in adaptive shrinking grid search (ASGS-CWOA) when the number of dimensions is 2; (**b**) the situation when the number of dimensions is 3, where the step size of migration is as follows: step_a = 10, or the step size of siege, step_c = 10; and the searching number is K = 2 (the red point means the current location of the wolf, while the black ones mean the searching locations of the wolf).

According to the principle of ASGS, during the process of migration or siege, the location of the current wolf is taken as the center of the search in D directions at the same time (here, D is the dimension of the solution space). The diagram can be seen in Figure 1.

From Figure 1, it is easy to see that a large number of points is distributed around the current wolf in some special way, and that means the best solution should be found in the local neighborhood space centered on the current wolf at the end of this process.

Generally, the strategy of ASGS enhances the performance of exploitation.

• Strategy of Opposite–Middle Raid (OMR)

The main idea of this is that the opposite location from the current wolf relative to the lead wolf should be inspected first; if the opposite wolf has better fitness than the current one, then the current wolf will move to the opposite one. Otherwise, 2D points (D means the dimension number of the solution space) around the current point and the middle point (regarding the current wolf and the leader wolf) should be inspected. Thus, the point with the best local fitness can be found, which becomes the new location of the current wolf at the end of this process.

In short, this enhances the performance of exploration.

• Adaptive Standard Deviation Updating Amount (ASDUA)

ASDUA is a dynamic number that reflects the dynamic situation of the wolf pack during any iteration, which means the population of the wolf pack and the standard deviation of its fitness determine how many wolves there are and who will be eliminated and regenerated. The standard deviation can be obtained by Equation (1):

(1) $$\sigma =\sqrt{\frac{1}{N}\sum_{i=1}^{N}{\left(x_{i}-\mu \right)}^{2}}\;,$$

where *N* means the size of the wolf pack; *x_i_* means the fitness of the *i*-th wolf; and *μ* is the mean value of fitness. Then, ASDUA is gained by the following formula.

(2) $$\mathrm{ASDUA}\{\begin{array}{l} \mathrm{ASDUA}+1,\;\mathrm{if}\;\mathrm{fitness}(\mathrm{xi})<(\mu -\sigma /2)\\ \mathrm{Do}\;\mathrm{nothing},\;\mathrm{if}\;\mathrm{fitness}(\mathrm{xi})\geq (\mu -\sigma /2) \end{array}$$

Aiming at the problem of solving the maximum, ASDUA is zero when the iteration begins; next, the difference between the mean value and the SD regarding the fitness of the wolf pack should be computed, and if the fitness of the current wolf is less than the difference, ASDUA increases by 1; otherwise, nothing changes. Thus, the value of ASDUA is obtained, and ASDUA wolves are eliminated and regenerated.

In brief, this enhances the performance of the exploration as well as the OMR.

### 2.2. Some Covering Models

Generally speaking, a wireless sensor network hopes to cover all the monitered area and include a few sensors that are as far apart as possible. Several classical covering models are listed as follows.

A: Inscribed Rectangular – Full Covering Model (IRFCM)

**Figure 2:**
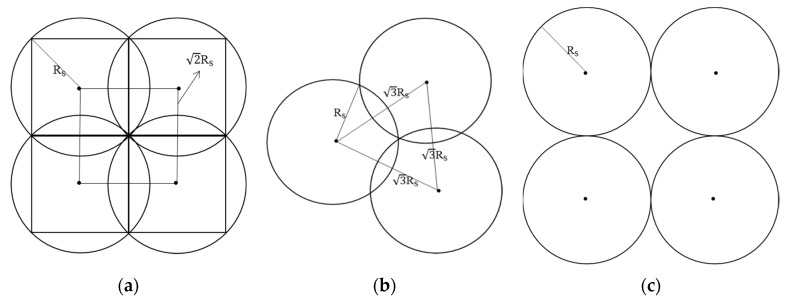
(**a**) The diagram of the inscribed rectangular full covering model; (**b**) the diagram of the minimum overlapping full covering model; and (**c**) the diagram of the tangent covering model of the adjacent boundary (the black point means the current location of the sensor, while the black circle means the boundary of the area sensed by the sensor).

As seen in Figure 2a, in IRFCM, the area of the maximum inscribed rectangle about the sensing circle is the effective covering area of the corresponding sensor, and it is under this model that the covering rate of the wireless sensor network (WSN) is high and is easy to expand; however, the overlapping rate is too high, and its value reaches (π − 2)/π (π means the circular constant). This model requires that Rc is greater than or equal to √2 ∗ Rs.

B: Minimum Overlapping Full Covering Model (MOFCM)

As seen in Figure 2b, in MOFCM, the area of the overlapping parts of any two intersecting circles can be calculated according to the radius and the distance between their centers, and the overlapping area is Rs2 ∗ (2 ∗ π − 3√3)/6. Thus, the overlapping rate is (2 ∗ π – 3√3)/(6 ∗ π), which is lower than the value (π − 2)/π from IRFCM, as detailed in Figure 2 (b). Under this model, the covering rate of the WSN is high, as under IRFCM, and it has the possibility to expand. This model requires that Rc is greater than or equal to √3 ∗ Rs. A detailed discussion can be found in [17].

C: Tangent Covering Model of Adjacent Boundary

As seen in Figure 2c, under this model, the overlapping rate is zero, which is the best state hoped for, while the coverage is unsatisfactory, in that there are obvious coverage vulnerabilities. This model requires that Rc is greater than or equal to 2 ∗ Rs.

### 2.3. Assumptions

Put simply, some assumptions are as follows regarding the characteristics of this paper.

A: The wireless sensor network should have an intelligent control and management center that can communicate with all wireless sensor nodes and obtain their positions by some methods, including the BEIDOU (Beidou Navigation Satellite System) and GPS (global positioning system), and so on. This intelligent center should own the map of the whole space which the wireless sensor network is intended to monitor.

B: The intelligent center should have optimized computing power in order to obtain the global optimal solution according to the methods in this paper.

C: Wireless sensor nodes should be movable and able to go to the designated location.

D: All the sensor nodes should have same the sensing radius Rs and communication radius Rc, centered on themselves respectively. The Euclidean distance is adopted in this paper.

## 3. Proposed Method

To adopt ASGS-CWOA with good performance to address the low coverage of WSN, we made improvements in the following aspects.

### 3.1. Adaptive Expansion Based on the Minimum Overlapping Full Covering Model (AE-MOFCM)

**Figure 3:**
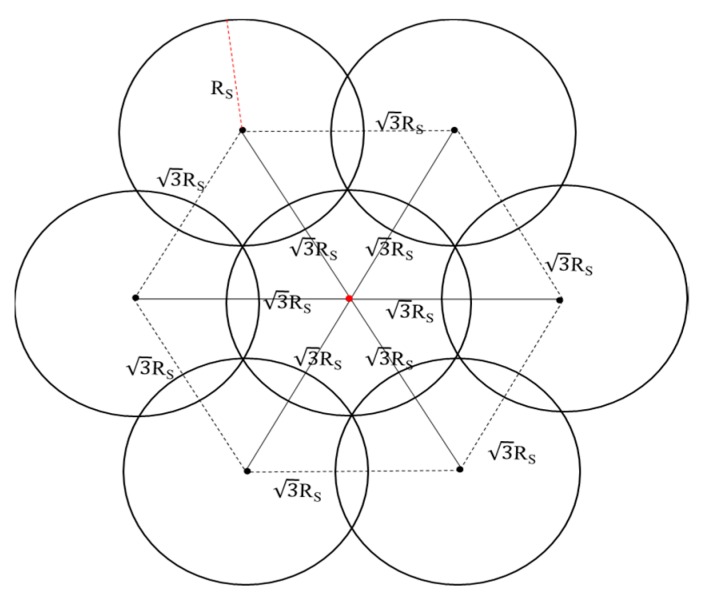
Diagram of the adaptive expansion based on the minimum overlapping full-coverage model (AE-MOFCM). The red point is a single given location; the red dashed line means the sensing radius of sensors, and the value is Rs; while the black points are subsequent points that can be obtained by the corresponding formula.

Compared to the other covering models mentioned above, MOFCM makes wireless sensor networks have high coverage and easy deployment as well as an acceptably low overlapping rate. Thus, this model is selected as the basic covering model for the WSN (Figure 3).

(3) $$Target_{1}=[\left(range_{max}-\;range_{min}\right)/2,\;\left(range_{max}-\;range_{min}\right)/2];$$

(4) $$\left\{ \begin{array}{l} Target_{i+1}=Target_{i}+\left[DISTANCE,\;0\right]\\ Target_{i+2}=Target_{i}+\left[DISTANCE/2,\;DISTANCE*\sqrt{3}/2\right]\\ Target_{i+3}=Target_{i}+\left[-DISTANCE/2,\;DISTANCE*\sqrt{3}/2\right]\\ Target_{i+4}=Target_{i}+\left[-DISTANCE,\;0\right]\\ Target_{i+5}=Target_{i}+\left[-DISTANCE/2,\;-DISTANCE*\sqrt{3}/2\right]\\ Target_{i+6}=Target_{i}+\left[DISTANCE/2,\;-DISTANCE*\sqrt{3}/2\right]\\ If\;Target_{i}\;\notin \left[range_{min},\;range_{max}\right],\;Target_{i}=Target_{i+1},\;(i\;=\;1,2,\ldots ,N_{Target}), \end{array}\right.$$

where *range_max_* and *range_min_* are the upper and lower limit of the solution space, respectively; *DISTANCE* means √3R, and R is the sensing radius of a single sensor; and *N_Target_* means the number of the target locations.

(5) $$\left\{ \begin{array}{ll} Assist_{Target}{}_{j}= & (Target_{current}+\left[R,0\right]),\;If\;(Target_{current}+\left[DISTANCE,0\right])\;\notin \\ & \left[range\_min,range\_max\;\right]\;and\;(Target_{current}+\left[R,0\right])\;\in \;\left[range\_min,range\_max\;\right]\\ Assist_{Target}{}_{j}= & \left(Target_{current}+\left[R\sqrt{2}/2,R\sqrt{2}/2\right]),\;If\;(Target_{current}+\left[DISTANCE/2,\;DISTANCE*\sqrt{3}/2\right]\right)\\ & \notin \;\left[range\_min,range\_max\;\right]\;and\;(Target_{current}+\left[R\sqrt{2}/2,R\sqrt{2}/2\right])\\ & \in \;\left[range\_min,range\_max\;\right]\\ Assist_{Target}{}_{j}= & (Target_{current}+\left[-R\sqrt{2}/2,R\sqrt{2}/2\right]),\;If\;(Target_{current}+\left[-DISTANCE/2,\;DISTANCE*\sqrt{3}/2\right)\\ & \notin \;\left[range\_min,range\_max\;\right]\;and\;(Target_{current}+\left[-R\sqrt{2}/2,R\sqrt{2}/2\right])\\ & \in \;\left[range\_min,range\_max\;\right]\\ Assist_{Target}{}_{j}= & \left(Target_{current}+\left[-R,0\right]),\;If\;(Target_{current}+\left[-DISTANCE,0\right]\right)\\ & \;\notin \;\left[range\_min,range\_max\;\right]\;and\;(Target_{current}+\left[-R,0\right])\;\in \;\left[range\_min,range\_max\;\right]\\ Assist_{Target}{}_{j}= & (Target_{current}+\left[-R\sqrt{2}/2,-R\sqrt{2}/2\right]),\;If\;(Target_{current}+[-DISTANCE/2,\;-DISTANCE\\ & *\sqrt{3}/2])\;\notin \;\left[range\_min,range\_max\;\right]\;and\;(Target_{current}+\left[-R\sqrt{2}/2,-R\sqrt{2}/2\right])\\ & \in \;\left[range\_min,range\_max\;\right]\\ Assist_{Target}{}_{j}= & (Target_{current}+\left[R\sqrt{2}/2,-R\sqrt{2}/2\right]),\;If\;(Target_{current}+[DISTANCE/2,\;-DISTANCE*\\ & \sqrt{3}/2])\;\notin \;\left[range\_min,range\_max\;\right]\;and\;(Target_{current}+\left[R\sqrt{2}/2,-R\sqrt{2}/2\right])\\ & \in \;\left[range\_min,range\_max\;\right]\\ & (j=1,2,3,\ldots ). \end{array}\right.$$

**Figure 4:**
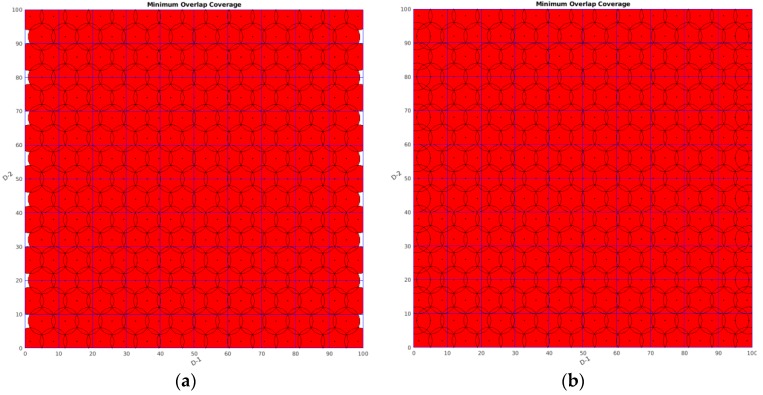
Here, rangemax = 100 and rangemin = 0, while the sensing radius of a single sensor is 4. The black point means a target location, the black circle means the sensing space boundary of a single sensor, and the red area means the whole sensing space of the wireless sensor network, while the very small white areas are vulnerabilities in monitoring. (**a**) The diagram without special treatment of the boundary and (**b**) with special treatment.

First of all, the first target can be generated by Equation (3), and then the others can be obtained by Equation (4). It is important to note in particular that the array of target locations should not take in the targets that are out of [rangemin, rangemax], which should be processed by another array according to Equation (5). Another array named AssistTarget-locations is related to special treatment of the boundary of the solution space. A demo diagram of the AE-MOFCM is shown in Figure 4.

### 3.2. Improvement of ASGS-CWOA

The adaptive shrinking grid search wolf pack optimization algorithm is oriented to the continuous solution space, and the path planning of WSN refers to the discrete solution space, which enables ASGS-CWOA to not be used directly for the coverage problem of WSN. Therefore, we proposed a new variant of the wolf pack optimization algorithm by absorbing some ideas of ASGS-CWOA and making some necessary improvements according to the special conditions of optimization problems oriented to discrete space. The new variant includes two improvements, which are detailed as follows.

#### 3.2.1. Target–Node Probability Matrix

N targets and N sensor nodes are in the WSN, meaning that according to the one-to-one correspondence between sensor nodes and target points, there are (N!) matching options. To choose the optimal, shortest overall path length, some matrices have been designed.

(6) $$Dis_{Matrix-k}=[Distance\left(target\left[k\right],node\left(1\right)\right),\;Distance\left(target\left[k\right],node\left(2\right)\right),\ldots ,\;Distance(target\left[k\right],node\left(N\_Node\right));$$

(7) $$Dis_{Total_{Reverse}-k}=\;\sum_{i=1}^{N\_Node}1/Dis_{Matrix-k}\left[i\right];$$

(8) $$Probability_{Target-k_{disperse}}=\left[1/Dis_{Matrix-k}\left[1\right],\;1/Dis_{Matrix-k}\left[2\right]\ldots ,1/Dis_{Matrix-k}\left[N\_Node\right]\right]/Dis_{Total_{Reverse}-k};$$

(9) $$Probability_{Target-k}\left[i\right]=\;\sum_{j=1}^{i}Probability_{Target-k_{disperse}}\left[j\right]\;(i=1,2,\ldots ,N\_Node);$$

(10) $$Probality_{Matrix}\left[k\right]=Target\left[k\right]-[Probability_{Target}\left[1\right],\;Probability_{Target}\left[2\right],\ldots ,\;Probability_{Target}\left[N\_Node\right]\;\;(k=1,2,\ldots ,N\_Node);$$

where *N_Node* is the number of wireless sensor nodes, and *N_Target* is the number of destinations that wireless sensor nodes will move to. *Dis_Matrix-k_* indicates the matrix consisting of the distances of each sensor node to the *k*-th target location. *Dis_Total_Reverse-k_* indicates the reciprocal sum of all elements of the distance matrix about the *k*-th target. *Probability_Target-k_disperse_* indicates the probability matrix consisting of the possibilities of each node moving to the *k*-th target location. *Probability_Matrix_*[*k*] is another expression of *Probability_Target-k_disperse_* with a continuous space between 0 and 1, and it is conducive to the use of Roulette for computing.

(11) $$Min-Dis_{Matrix}=sort(Dis_{Matrix},\;ascend),$$

where *Min-Dis_Matrix_* means the ascending index arranged according to values of $Dis_{Matrix}$; *sort(Dis_Matrix_, ascend)* will return an index, which is arranged as ascending according to values of $Dis_{Matrix}$.

In order to describe the practical application of the target–node probability matrix in the wolf pack optimization algorithm, an example is shown as follows. 

Put simply, the range of the solution space is set as [0, 100], and the sensing radius of a sensor is 30. Thus, the targets can be obtained by AE-MOFCM, and the sensors can be obtained randomly by Equations (13) and (14).

Targets = (50, 50); (75.9808, 95); (24.0192, 95); (24.0192, 5); (75.9808, 5); (80, 50); (20, 50); (97.1940, 73.7868); (2.8060, 73.7868); (2.8060, 26.2132); (97.1940, 26.2132).

Sensors = (9.4925, 68.6594); (77.9807, 98.643); (18.8638, 98.5761); (0.9487, 45.6081); (41.0077, 34.8011); (14.1326, 98.2914); (53.2759, 48.587); (55.776, 31.5094); (69.344, 38.1385); (87.6369, 1.801); (1.9635, 50.3632).

The *Dis_Matrix_* can be obtained by Equation (6), *Probability_Target-Disperse_* can be obtained by Equations (7) and (8), *Probability_Target_* can be obtained by Equations (9) and (10), and *Min-Dis_Matrix_* can be obtained by Equation (11), detailed as follows.

*Dis_Matrix_* =43.778951.152023.417248.564441.008845.226943.737159.080722.802352.457552.683789.5848102.795534.116496.670582.339096.447658.4000110.912465.449057.799075.208194.501193.070357.392169.963450.295377.626214.684493.442643.79376.590628.757034.75258.132573.026027.781644.539213.740876.25828.916046.436286.964772.766017.399044.395656.593972.264378.954758.865294.922360.411467.2458104.2120100.619047.896667.943314.786976.340670.961469.548968.246180.322452.780474.535180.397657.784949.160751.750126.251011.310732.463831.118248.26357.356441.810329.788976.998797.013229.4629101.406592.082796.731478.2804108.686773.475480.946093.669687.021577.145668.826346.896827.548858.357318.006872.992133.187123.72241.263159.080837.518876.25514.458721.688517.892757.308027.466735.034567.360948.614042.972169.769544.111690.021490.502479.193095.329985.084574.3281102.6011105.5259

*Probability_Target-Disperse_* =0.03970.03160.03070.03600.10030.02950.49670.09150.07810.02900.03690.03610.62050.04510.02870.03700.04160.04990.03870.04500.02750.02980.07980.04440.38270.04410.03840.23050.04380.03380.03300.02130.04820.07930.04790.05530.11090.15100.05520.09870.12520.09230.08130.10270.04390.04320.03690.04740.08800.03610.08230.12130.11970.33460.04660.05230.07840.04890.04820.09120.04670.14260.12520.23930.07820.04890.14030.03970.06180.15370.11590.06180.09020.07460.05920.03620.16660.06530.18270.06990.05720.08390.06630.11330.09700.12690.07900.05850.33590.03570.09580.10020.05190.10480.05020.04180.03750.02540.12080.09640.03970.05590.21270.10580.05680.07510.07780.06130.04690.17150.05250.06830.04800.05210.09000.04650.10380.12250.16890.19520.0521

*Probability_Target_* =0.03970.07130.1020.1380.23840.26780.76450.8560.93410.96311.00000.03610.65660.70170.73040.76740.8090.8590.89770.94270.97021.00000.07980.12420.5070.5510.58940.81990.86360.89750.93050.95181.00000.07930.12730.18260.29350.44450.49970.59840.72370.8160.89731.00000.04390.08710.1240.17140.25940.29550.37780.49920.61880.95341.00000.05230.13070.17960.22780.3190.36570.50830.63350.87290.95111.00000.14030.180.24190.39560.51140.57320.66340.7380.79730.83341.00000.06530.2480.31790.37510.4590.52530.63860.73560.86240.94151.00000.33590.37160.46750.56770.61950.72440.77460.81630.85380.87921.00000.09640.13610.1920.40470.51060.56740.64240.72030.78160.82851.00000.05250.12080.16880.22090.31090.35750.46130.58380.75270.94791.0000

*Min-Dis_Matrix_* =75891114236102739685111410361112475891058411791013621089574111236978521011134611415783692102978510361114111643578921041151879631021098752141136

#### 3.2.2. Adaptive Step Size

(12) $$\left\{ \begin{array}{l} stepa\;=\;stepa_{basic}*\;\left(1/ceil\left(3*t/T\right)\right),\;stepa_{basic}=ceil\left(N_{Node}/2\right)\\ stepb\;=\;stepb_{basic}\;*\;\left(1/ceil\left(3*t/T\right)\right),\;stepb_{basic}\;=ceil\left(N_{Node}/3\right)\\ stepc\;=\;stepc_{basic}\;*\;ceil\left(3*t/T\right),\;stepc_{basic}\;=ceil(N_{Node}/3), \end{array}\right.$$

where *stepa_basic_* is the original step size of migration, *stepb_basic_* is the step size of summon, and *stepc_basic_* is the step size of siege; *t* is the number of the current iteration, and *T* means the maximum number of iterations; and *ceil*() is a function that can round a number up.

During the early stage of optimization, for migration, it is hoped that a search for large areas can be conducted to find potential global optima, while the search for small areas should be conducted to accelerate the speed of convergence in the later stage.

For summon, it is in the former stage that a larger step size is needed to enable other wolves to move quickly towards the location of the best wolf, so that the rate of convergence can be enhanced, while a smaller step size can stop the algorithm from falling into a local optimum in the later stage.

Finally, for siege, a smaller step size makes the algorithm converge quickly in the early stage, while a larger one can prevent the algorithm from falling into the local optimum in the later stage. 

## 4. Main Steps

In order to clearly show the details of the new proposed algorithm, specific implementation steps are given as follows.

Step 1: Initialization

Initially, all the movable sensor nodes will be randomly and unorderly scattered in the monitored area, just as wolves are distributed randomly and unorderly in the solution space. Thus, the locations of sensor nodes can be represented by the wolf population generated by Equation (13).

w*i* = (w*i*1, w*i*2, …, w*id*, …, w*i*D)(*i* = 1, 2, …, N; d = 1, 2, …, D), (13)

where wid is the location of the *i*-th wolf in the *d*-th dimension. N means the size of the wolf population. D is the maximum dimension number of the solution space. The initial location of each wolf can be produced by Equation (14).

w*id* = rangemin + rand(0,1)*(rangemax – rangemin), (14)

where rand (0, 1) is a random number distributed uniformly in the interval [0, 1]; rangemax and rangemin are the upper and lower limits of the solution space, respectively. 

Step 2: Migration

In this process, each wolf takes its own location as the center to search and moves along D directions, meaning that new locations will be generated, and the best one should be the destination that the current wolf is devoted to. The formula is as follows:

(15) $$wolf_{new}=[wolf\left(1\right),wolf\left(2\right),\ldots \;wolf\left(RAND\right),\ldots ,\;wolf\left(N\_Node\right)];$$

(16) $$\left\{ \begin{array}{ll} wolf\left(RAND\right)= & i,Probability_{Matrix}[RAND][i-1]<rand\left(1,1\right)<\\ & Probability_{Matrix}[RAND][i],(i=2,3,\ldots ,N\_Node)\\ wolf\left(RAND\right)= & 1,\;rand\left(1,1\right)<Probability_{Matrix}\left[RAND\right]\left[1\right]; \end{array}\right.$$

where *RAND* is a randomly generated positive integer between 1 and *N_Node*, and *rand*(1,1) generates a random number between 0 and 1. 

If the new wolf has better fitness than the current wolf, the latter should move to the new location and update the corresponding fitness. Otherwise, it continues to loop until the number is out of *stepa*, which is the step size of migration and is obtained by Equation (12).

Step 3: Summon–Raid

(17) $$\left\{ \begin{array}{ll} {wolf}_{new}= & [wolf(1),wolf(2),\ldots wolf\left(index(i)\right)\ldots ,wolf(RAND-1),bestwolf(RAND),bestwolf(RAND+1),\\ & \ldots bestwolf(RAND+stepb-1),wolf(RAND+stepb),\ldots wolf(N_{Node}))\\ wolf\left(index\right)= & \;bestwolf\left(RAND:\;\left(RAND\;+\;stepb\;-1\right)\right),\;\;index\left(i\right)\notin \left[RAND,\;\left(RAND+\;stepb\;-1\right)\right]\\ & \left(RAND<=\;N_{Node}–\;stepb\;+\;1\right),\left(i=1,2,\ldots ,stepb\right)\; \end{array}\right.$$

The current wolf moves towards the lead wolf with the best fitness with the step size “*stepb*” according to Equation (17); if the fitness of the new location is better than the current one, the wolf should move to the new location and update the corresponding fitness. Otherwise, it stays at the old position; here, *stepb* means the step size of summon–raid, which can be obtained by Equation (12).

Step 4: Siege

(18) $$\left\{ \begin{array}{l} wolf_{new}=\left[wolf\left(1\right),\;wolf\left(2\right),\ldots ,wolf\left(i\right),\;\ldots ,wolf\left(N_{Node}\right)\right]\;\;\;\;\;\;\;\left(i=1,2,\ldots ,N_{Node}\right)\\ wolf\left(i\right)=Min-Dis_{Matrix\left[i\right]\left[j\right]}\;\;\;\;\;\;\;\;\;\;\;\;\;\left(j=1,2,\ldots ,stepc\right) \end{array}\right.$$

During this process, each wolf performs small-scale, fine-grained searches for potential global optima; that is, for each dimension of each wolf, several searches will be conducted according to the step size *stepc* (which can be obtained by Equation (12)) and a shorter distance matrix, whose name is *Min-Dis_Matrix_*, as stated above. The new location to which the current wolf tries to move to can be obtained by Equation (18), and if the fitness of the new location is better than the current fitness, the current wolf should move to the new position; otherwise, it stays at the old one. At the end of this process, the new wolf has a new fitness that is no less than its original fitness.

Step 5: Updating Population

After siege, some wolves with poorer fitness will be eliminated, while the same number of wolves will be regenerated, and this amount can be obtained by Equation (2).

**Figure 5:**
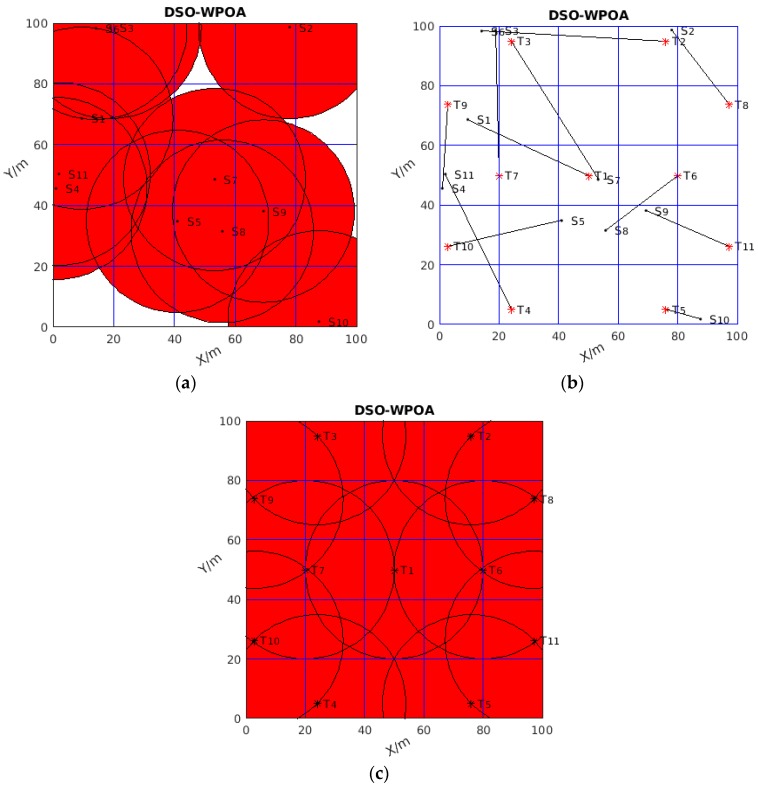
(**a**) The initial diagram of wireless sensor network coverage (distributed randomly); (**b**) the solution of how the movable sensors move to the targets one by one; and (**c**) the coverage that has been optimized by DSO-WPOA The red stars mean the locations of targets, while the black points mean the randomly generated initial locations of sensors, and the black lines mean the moving paths. Specifically, the parameters in the diagram correspond to the example in Section 3.

Across the whole process of this newly proposed algorithm, the purpose is to find the shortest path under a high coverage rate to reduce energy consumption and improve the sustainability of the whole network as much as possible. The process is shown briefly in Figure 5.

## 5. Experiments

To evaluate the performance of the proposed algorithm, several comparative experiments were conducted with the Ubuntu 16.04.4 operating system, Intel(R) Core(TM) i7-5930K processor, 64G memory and MATLAB-2017b. We also used algorithms such as the random algorithm, virtual force algorithm (VFA), and coverage algorithm based on particle swarm optimization (PSO-WSN). In this paper, the range of the solution space was [0,1000]; for all related algorithms, the number of sensors was 77, the sensing radius of a sensor was 80, and the communication radius of sensors was more than 80*√3. The PSO-WSN was conducted based on the idea behind the paper [18], while the VFA was based on the basic concept of VFA [10,11,26,27], and the steps to implement DSO-WPOA followed those described in Section 4. Furthermore, the main parameters of DSO-WPOA were the following: the size of the wolf population was 50, the initial step size of migration was stepa = 39, the initial step size of summon–raid was stepb = 26, and the step size of siege was stepc = 26.

**Figure 6:**
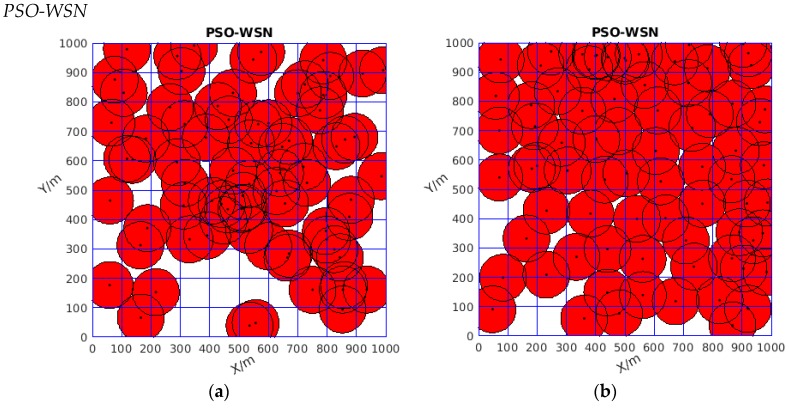
(**a**) The initial diagram of wireless sensor network coverage (distributed randomly); (**b**) the coverage optimized by the PSO.

**Figure 7:**
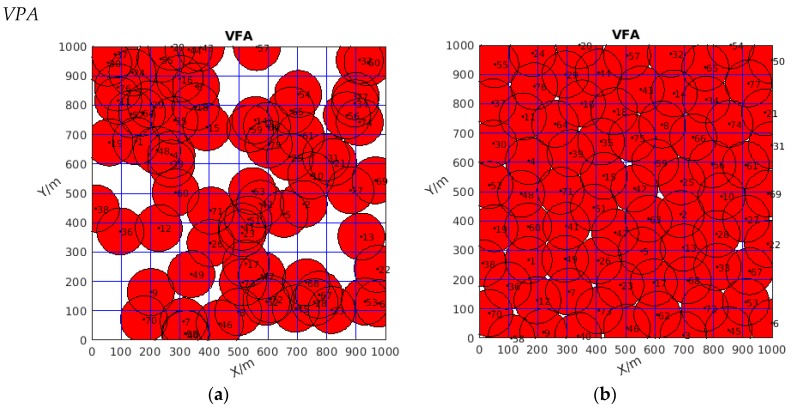
(**a**) The initial diagram of wireless sensor network coverage (distributed randomly); (**b**) the coverage optimized by VPA.

**Figure 8:**
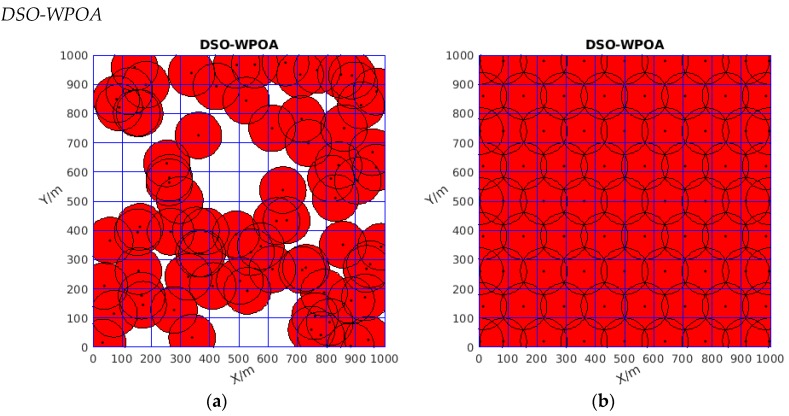
(**a**) The initial diagram of wireless sensor network coverage (distributed randomly); (**b**) the coverage optimized by DSO-WPOA.

Firstly, focusing on Table 1, seen from the perspective of the best coverage rate, the value of the new algorithm could reach 100%, which means all the monitoring area was covered. Although the two other algorithms had good coverage rates that reached 93.83% and 99.645%, respectively, some white area existed in the monitoring area, as shown in Figure 6b and Figure 7b, which means that some area was uncovered, while no white gap existed in Figure 8b. Thus, DSO-WPOA possessed the best coverage rate.

Moreover, after repeating 100 times, both the worst and mean coverage rates of DSO-WPOA reached 100%, and its variance was zero, while the terms of the two other algorithms were less than the former. Therefore, although the variance of VFA was very small, DSO-WPOA had better stability in general.

Furthermore, focusing on the mean time, the PSO-WSN required the most time, and the time the newly proposed algorithm required was on the same order of magnitude as that of VFA. Thus, DSO-WPOA possessed an acceptable time performance.

Finally, the mean moving distance according to the rules of DSO-WPOA was smallest; the value was 7,662,2987, while the values of the other two algorithms were 3,6575,491 and 1,1094,4339, respectively, which means the new algorithm enabled the WSN to spend less energy. Consequently, DSO-WPOA possessed advantages in saving energy.

In summary, DSO-WPOA had the best coverage rate, better stability, acceptable performance in terms of time, and advantages in saving energy.

## 6. Discussion and Conclusions

To further improve the coverage rate of a movable wireless sensor network under the condition of guaranteed connectivity, we proposed an adaptive, discrete space oriented wolf pack optimization algorithm for a movable wireless sensor network. Firstly, a strategy of adaptive expansion based on the minimum overlapping full-coverage Model was designed to achieve minimum overlap and no-gap coverage of the monitoring area. Moreover, the ASGS-CWOA was improved to optimize the movable wireless sensor network, which is a discrete space oriented problem, and the improvement included the usage of a target–node probability matrix and the design of an adaptive step size method, both of which worked together to improve the convergence speed and global optimization ability of the algorithm.

Theoretical research and experimental results indicated that, compared to the PSO-WSN algorithm and classical virtual force algorithm, the newly proposed algorithm, named DSO-WPOA, possessed the best coverage rate, better stability, acceptable performance in terms of time, advantages in energy saving, and no gaps. As detailed in Table 1, Figure 4b, and Figure 8b, the newly proposed algorithm enabled the WSN to cover the whole monitored area (no gaps) with the smallest number of sensors as distant as possible from each other. However, it performed weaker in some aspects; for example, DSO-WPOA spent more time on iterations than VFA, as shown in Table 1. The coverage rate of DSO-WPOA may not be superior to other coverage algorithms when not enough sensor nodes can be supplied, such as in this paper where the number of sensors was 77, which just satisfied AE-MOFCM. Assuming that the number of sensors is less than 77, the advantages of DSO-WPOA may be gone.

Our future work will continue to perfect the performance of DSO-WPOA in all aspects and apply it to specific projects as well to expand its scope of application.

## Figures and Tables

**Table 1:** Results of experiments. PSO-WNS: particle swarm optimization-wireless sensor network; VFA: virtual force algorithm.

Algorithm	Initial Coverage Rate	Best Coverage Rate	Mean Coverage Rate	Worst Coverage Rate	Variance	Mean Time	Moving Distance
PSO-WSN	77.71%	93.83%	91.228%	89.74%	12593.36	2124.0355	36575.491
VFA	76.10%	99.645%	99.476%	98.149%	1.7324e-06	23.2982	11094.4339
DSO-WPOA	76.53%	100%	100%	100%	0	57.8892	7662.2987

## References

[1] Kumar D.P., Amgoth T., Annavarapu C.S.R. (2019). Machine learning algorithms for wireless sensor networks: A survey. Inf. Fusion.

[2] Dai H.-N., Wong R.C.W., Wang H. (2017). On Capacity and Delay of Multichannel Wireless Networks With Infrastructure Support. IEEE Trans. Veh. Technol..

[3] Dai H., Wong Raymond C., Wang H. (2019). Big Data Analytics for Large ScaleWireless Networks: Challenges and Opportunities. ACM Comput. Surv..

[4] Thakur D., Kumar Y., Kumar A., Singh P.K. (2019). Applicability of Wireless Sensor Networks in Precision Agriculture: A Review. Wirel. Pers. Commun..

[5] Li G., Zhao J., Wang Z. (2006). Forest Fire Detection System based on Wireless Sensor Network. Chin. J. Sens. Actuators.

[6] Xu L., Nie J., Ma X., Lv H., Yang J. (2018). Design and Implementation of Water Environment Monitoring System Based on Wireless Sensor Network. Digit. Technol. Appl..

[7] Fu X., Yao H., Postolache O., Yang Y. (2019). Message forwarding for WSN-Assisted Opportunistic Network in disaster scenarios. J. Netw. Comput. Appl..

[8] Yick J., Bharathidasan A., Pasternack G., Mukherjee B., Ghosal D. Optimizing Placement of Beacons and Data Loggers in a Sensor Network—A Case Study. Proceedings of the IEEE Wireless Communications and Networking Conference (IEEE Cat. No.04TH8733).

[9] Qu Y., Zhai Y., Lin Z., Zhao B., Zhang Y. (2004). A Novel Sensor Placement Model in Wireless Sensor Network. J. Beijing Univ. Posts Telecommun. Telecommun..

[10] Zhou F., Gao J., Fan X., An K. (2018). Dynamic Covering Algorithm of Node Based on Virtual Force in Wireless Sensor Networks. J. Syst. Simul..

[11] Zhang C. (2019). Coverage Algorithm based on Virtual Forces in Wireless Sensor Networks. Appl. Res. Comput..

[12] Yarinezhad R., Hashemi S.N. (2019). A routing algorithm for wireless sensor networks based on clustering and an fpt-approximation algorithm. J. Syst. Softw..

[13] Chen Y., Xu X., Wang Y. (2019). Wireless sensor network energy efficient coverage method based on intelligent optimization algorithm. Discret. Contin. Dyn. Syst. S.

[14] Somaieh Z., Shahram B. (2019). Dehcic: A Distributed Energy-aware Hexagon based Clustering Algorithm to Improve Coverage in Wireless Sensor Networks. Peer Peer Netw. Appl..

[15] Alavi S.A., Mehran K., Hao Y., Rahimian A., Mirsaeedi H., Vahidinasab V. (2019). A Distributed Event-Triggered Control Strategy for DC Microgrids Based on Publish-Subscribe Model over Industrial Wireless Sensor Networks. IEEE Trans. Smart Grid.

[16] Sajwan M., Gosain D., Sharma A.K. (2018). CAMP: Cluster aided multi-path routing protocol for wireless sensor networks. Wirel. Netw..

[17] So-In C., Nguyen T., Nguyen N. (2019). An Efficient Coverage Hole-Healing Algorithm for Area-Coverage Improvements in Mobile Sensor Networks. Peer-Peer Netw. Appl..

[18] Wang X., Wang S., Ma J. (2007). Parallel Particle Swarm Optimization based Mobile Sensor Node Deployment in Wireless Sensor Networks. Chin. J. Comput..

[19] Yang C., Tu X., Chen J. Algorithm of Marriage in Honey Bees Optimization Based on the Wolf Pack Search. Proceedings of the International Conference on Intelligent Pervasive Computing (IPC 2007), Institute of Electrical and Electronics Engineers (IEEE).

[20] Wang D., Qian X., Liu K., Ban X., Guan X. (2018). An Adaptive Distributed Size Wolf Pack Optimization Algorithm Using Strategy of Jumping for Raid (September 2018). IEEE Access.

[21] Huang H., Ren Z., Wei J. (2019). Improved Wolf Group Algorithm for Solving Traveling Salesman Problem. Appl. Res. Comput..

[22] Wu H., Zhang F., Li H., Liang X. (2015). Discrete Wolf Pack Algorithm for Traveling Salesman Problem. Control Decis..

[23] Martin B., Marot J., Bourennane S. Improved Discrete Grey Wolf Optimizer. Proceedings of the 26th European Signal Processing Conference (EUSIPCO).

[24] Wu R., Wang S. Discrete Wolf Pack Search Algorithm based Transit Network Design. Proceedings of the 7th IEEE International Conference on Software Engineering and Service Science (ICSESS).

[25] Wang D., Qian X., Liu K., Ban X. (2018). An Adaptive Shrinking Grid Search Chaos Wolf Optimization Algorithm with Adaptive Standard-Deviation Updating Amount. IEEE Access.

[26] Liu Z., Liu Q., Yuan Y., Guan X. (2013). Location Scheme in Wireless Sensor Networks based on Bayesian Estimation, Virtual Force and Genetic Algorithm. Control Decis..

[27] Li M., Shi W. (2011). Virtual Force-Directed Differential Evolution Algorithm based Coverage Enhancing Algorithm for Heterogeneous Mobile Sensor Networks. Chin. J. Sci. Instrum..

